# Co-targeting of Tiam1/Rac1 and Notch ameliorates chemoresistance against doxorubicin in a biomimetic 3D lymphoma model

**DOI:** 10.18632/oncotarget.23156

**Published:** 2017-12-08

**Authors:** Muhammad Ikram, Yeseon Lim, Sun-Yong Baek, Songwan Jin, Young Hun Jeong, Jong-Young Kwak, Sik Yoon

**Affiliations:** ^1^ Department of Anatomy, Pusan National University School of Medicine, Yangsan 50612, Korea; ^2^ Department of Mechanical Engineering, Korea Polytechnic University, Siheung 15073, Korea; ^3^ Department of Mechanical Engineering, Kyungpook National University, Daegu 41566, Korea; ^4^ Department of Pharmacology, Ajou University School of Medicine, Suwon 16499, Korea

**Keywords:** tiam1, notch, chemoresistance, doxorubicin, 3D lymphoma model

## Abstract

Lymphoma is a heterogeneous disease with a highly variable clinical course and prognosis. Improving the prognosis for patients with relapsed and treatment-resistant lymphoma remains challenging. Current *in vitro* drug testing models based on 2D cell culture lack natural tissue-like structural organization and result in disappointing clinical outcomes. The development of efficient drug testing models using 3D cell culture that more accurately reflects *in vivo* behaviors is vital. Our aim was to establish an *in vitro* 3D lymphoma model that can imitate the *in vivo* 3D lymphoma microenvironment. Using this model, we explored strategies to enhance chemosensitivity to doxorubicin, an important chemotherapeutic drug widely used for the treatment of hematological malignancies. Lymphoma cells grown in this model exhibited excellent biomimetic properties compared to conventional 2D culture including (1) enhanced chemotherapy resistance, (2) suppressed rate of apoptosis, (3) upregulated expression of drug resistance genes (MDR1, MRP1, BCRP and HIF-1α), (4) elevated levels of tumor aggressiveness factors including Notch (Notch-1, -2, -3, and -4) and its downstream molecules (Hes-1 and Hey-1), VEGF and MMPs (MMP-2 and MMP-9), and (5) enrichment of a lymphoma stem cell population. Tiam1, a potential biomarker of tumor progression, metastasis, and chemoresistance, was activated in our 3D lymphoma model. Remarkably, we identified two synergistic therapeutic oncotargets, Tiam1 and Notch, as a strategy to combat resistance against doxorubicin in EL4 T and A20 B lymphoma. Therefore, our data suggest that our 3D lymphoma model is a promising *in vitro* research platform for studying lymphoma biology and therapeutic approaches.

## INTRODUCTION

Lymphoma is the most common blood cancer. The incidence of malignant lymphoma around the world has been increasing at a rate of 3–4% over the last four decades. This is most likely due to improved diagnostic techniques, growth and aging of the world population, the recent trends in the acquired immune deficiency syndrome (AIDS) epidemic, and the rising number of cancer-causing behaviors although the reasons behind this lymphoma epidemic are still poorly understood [1]. Despite a better understanding of the biology of lymphoma and many important therapeutic advances in lymphoma treatments in the past decade, improving the prognosis for patients with relapsed and treatment-resistant disease remains an important challenge [2]. In particular, a major hurdle on the road to the discovery and development of new drugs is the lack of adequate testing models. Furthermore, one of the most important steps in developing a potential therapeutic drug is target identification.

Conventional 2-dimensional (2D) cell culture models do not accurately reflect the true biologic responses of cells in the human body where cells exist in a 3-dimensional (3D) microenvironment and cell-cell/cell-extracellular matrix (ECM) interactions occur through biochemical and mechanical cues [3–4]. Therefore, current *in vitro* drug testing models based on 2D cell culture systems result in disappointing clinical outcomes, and the invention of more efficient drug testing models using 3D cell culture systems is indispensable for the development of new drugs. Recent advances in cell biology, microfabrication techniques, and tissue engineering have enabled the development of a wide range of 3D cell culture technologies including multicellular spheroids, organoids, scaffolds, hydrogels, organs-on-chips, and 3D bioprinting, which are potentially useful to restore the morphological, functional, and microenvironmental features of human tissues and organs [5]. We recently reported the fabrication of a novel, physically gelated, bioactive, alginate/marine collagen/agarose (AmCA) composite hydrogel as a valuable matrix for biomimetic 3D cell spheroid formation and proposed its ability to efficiently create 3D multicellular spheroids for lymphoma cells [6].

Doxorubicin is one of the most important chemotherapeutic drugs, and it is widely used for the treatment of various types of tumors including hematological malignancies [7]. However, resistance to doxorubicin is a major obstacle to its clinical utility resulting in treatment failures, recurrences, and the need for high-dose therapy. Thus, in recent years, a great deal of attention has been paid to the development of strategies to circumvent its resistance mechanisms. It has been found that protein phosphatase 2A (PP2A), which is a key tumor suppressor; cyclosporine A, which is a modifier of multidrug resistance; and anti-multidrug resistance protein 1 (anti-MDR1) hammerhead ribozymes, which are modulators of MDR1-mediated drug resistance, potentiate the anticancer activity of doxorubicin in experimental hepatocellular carcinoma models [8–10]. To date, however, none of the studies has demonstrated benefits in clinical trials.

The Notch signaling pathway, a highly conserved cell signaling system present in most multicellular organisms, plays pivotal roles in regulating many cellular processes such as proliferation, survival, apoptosis, stem cell renewal and maintenance, cell fate specification, and differentiation [11]. Moreover, dysregulated Notch signaling is responsible for the development and progression of a wide range of human malignancies, including both solid tumors and hematologic malignancies [12–14]. Recently, it has been shown that the Notch pathway is also involved in drug resistance to tumor therapy [15]. Thus, in recent years much attention has been focused on Notch as a potential therapeutic target for the treatment of tumors by overcoming drug resistance of tumor cells and tumor recurrence [13, 16].

T-cell lymphoma invasion and metastasis 1 (Tiam1), a Rac1-specific guanine nucleotide exchange factor, was first identified as an invasion and metastasis-related gene [17]. Aberrant expression or mutations of Tiam1 has been shown to be associated with a variety of human cancer types including extranodal NK/T-cell lymphoma and chronic lymphocytic leukemia [18, 19]. Tiam1/Rac1 signaling is critically involved in tumor cell progression, invasion, and metastasis [13, 20]. Furthermore, it was shown that multidrug-resistant lymphoma cell lines express a higher Tiam1 level compared to multidrug-sensitive lymphoma cell lines [21]. Recently, it has been demonstrated that targeting Tiam1/Rac1 by using Tiam1 siRNA or inhibitors can reduce the chemoresistance in the proliferative and resistant pool of chronic lymphocytic leukemia (CLL) cells, which is considered to be associated with their recurrent relapses [22].

Thus, the aim of this work was to establish an effective *in vitro* 3D lymphoma model and to develop an efficient strategy to enhance chemosensitivity to doxorubicin using a new bioactive matrix for 3D cell culture, AmCA composite hydrogel. Importantly, we report that the combined inhibition of dual oncotargets, Tiam1 and Notch, could be a new therapeutic approach to overcome the resistance of EL4 T and A20 B lymphoma cells against doxorubicin.

## RESULTS

### 3D microenvironment in AmCA hydrogels promotes resistance against antitumor agents for lymphoma

**Figure 1:**
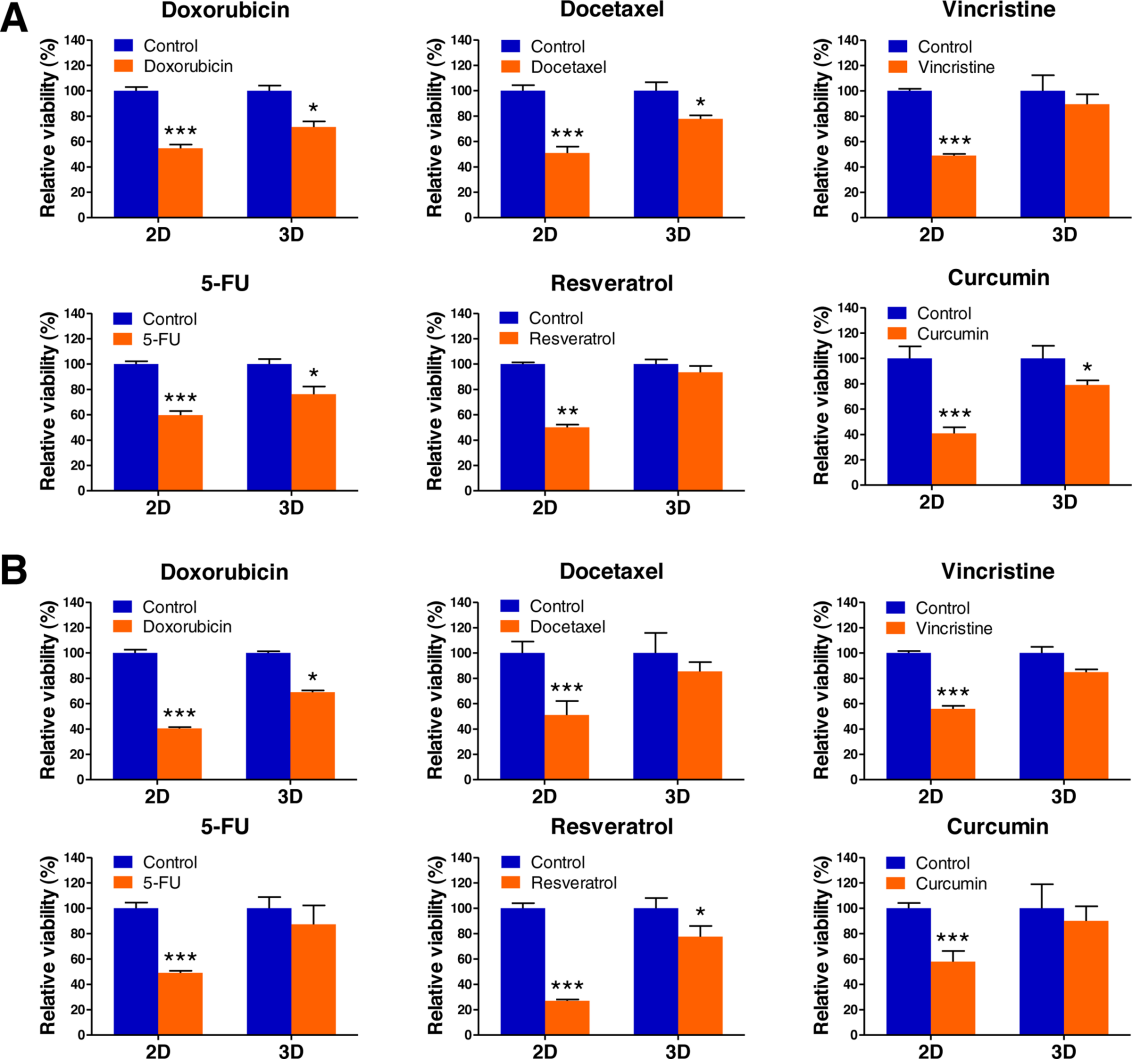
Lymphoma cell responses to chemotherapeutics in 2D and 3D cultures EL4 (**A**) and A20 (**B**) lymphoma cell viabilities in 2D and 3D culture after exposure to doxorubicin, docetaxel, vincristine, 5-FU, resveratrol, and curcumin. Data represent the mean percentage viability ± SD of three independent experiments normalized against untreated control cells. ^*^P < 0.05, ^**^P < 0.01, and ^***^P < 0.001 versus the control.

As tumor cells grown in 3D models that accurately reflect the 3D nature of the *in vivo* microenvironment are considered to exhibit a higher level of drug resistance over those in traditional 2D monolayer, we hypothesized that the 3D multicellular lymphoma spheroids grown within AmCA hydrogels may exhibit enhanced chemotherapeutic resistance to antitumor agents for lymphoma compared to cells cultured in 2D. To evaluate effects of the 3D microenvironment provided by AmCA hydrogels on drug resistance against various antitumor agents for lymphoma, we seeded EL4 or A20 cells at 1 × 10^5^ cells/ml into these hydrogels. On day 7, when multicellular spheroids were formed in 3D conditions, the cells grown in 2D or 3D cultures were exposed to 1 μM doxorubicin, 0.5 μM docetaxel, 1 μM vincristine, 5 μM, 5-fluorouracil (5-FU), 100 μM resveratrol, and 10 μM curcumin for 24 h. A WST-1-based colorimetric cell cytotoxicity assay was employed to evaluate the ability of each antitumor agent to induce cytotoxicity. A cell viability assay showed a significant difference between 2D and 3D cultures with regard to antitumor agent sensitivity. In EL4 cells, the rates of resistance in 3D cultures to doxorubicin, docetaxel, vincristine, 5-FU, resveratrol, and curcumin were 71.4 ± 4.4% (versus 54.6 ± 2.8% for 2D), 77.7 ± 2.7% (versus 50.9 ± 4.9% for 2D), 89.4 ± 7.8 (versus 48.9 ± 1.2% for 2D), 76.1 ± 6.1% (versus 59.6 ± 3.3% for 2D), 93.4 ± 5.1% (versus 50.1 ± 2.0% for 2D), and 78.9 ± 3.6% (versus 40.9 ± 4.8% for 2D), respectively (Figure 1A). An untreated condition (considered as 100%) grown during the same time served as the control. A similar effect was also seen with A20 cell spheroids, the rates of resistance in 3D cultures to doxorubicin, docetaxel, vincristine, 5-FU, resveratrol, and curcumin were 69.0 ± 1.3% (versus 40.4 ± 1.0% for 2D), 85.4 ± 7.3% (versus 51.0 ± 11% for 2D), 84.8 ± 2.2 (versus 55.8 ± 2.3% for 2D), 87.3 ± 1.94% (versus 49.0 ± 1.6% for 2D), 77.6 ± 8.3% (versus 27.0 ± 1% for 2D), and 90.0 ± 11.4% (versus 57.9 ± 8.3% for 2D), respectively (Figure 1B). These data demonstrate that the antitumor agent resistance of both cell types was robustly augmented in 3D cultures compared to 2D cultures.

As tumor cells grown in 3D models that adequately reflect the 3D nature of the *in vivo* microenvironment are thought to be more resistant to apoptosis induced by antitumor agents than those in traditional 2D monolayer cultures, we hypothesized that the multicellular 3D lymphoma spheroids grown within AmCA hydrogels may be less vulnerable to induction of apoptosis by antitumor agents than cells cultured in 2D were. To evaluate the effects of the 3D microenvironment provided by AmCA hydrogels on the susceptibility to apoptosis caused by doxorubicin, a representative antitumor agent for lymphoma, we seeded EL4 or A20 cells at 1 × 10^5^ cells/ml into these hydrogels. On day 7, when multicellular spheroids were formed in 3D conditions, the cells grown in 2D or 3D cultures were exposed to 1 μM doxorubicin for 24 h. To determine the mechanism employed in the regulation of apoptosis in the 3D multicellular spheroids, we examined the level of expression of antiapoptotic (Bcl-2 and Bcl-xL) and proapoptotic (Bax) proteins on day 10.

**Figure 2:**
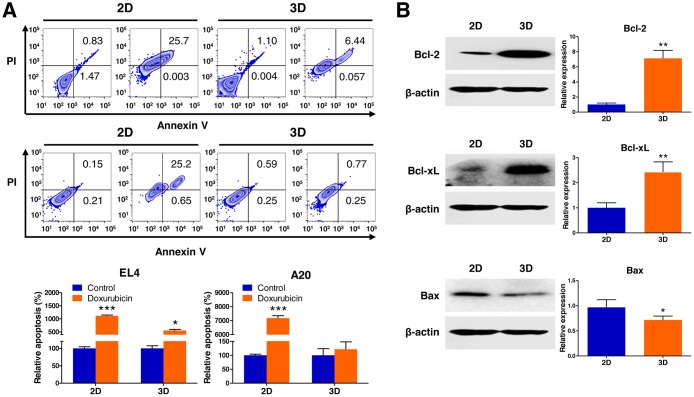
Analysis of apoptotic state in 3D lymphoma spheroids compared to their 2D cultures (**A**) Flow cytometric analysis demonstrates increased resistance to apoptosis of EL4 and A20 cells in response to treatment with 1 μM doxorubicin (Annexin V positive: apoptotic cells; PI positive: dead cells) in 3D spheroids compared to 2D culture. Apoptosis ratios were calculated from three independent experiments for both cell lines. (**B**) Western blot analysis of EL4 cells for the expression of apoptosis-regulating proteins in 2D and 3D cultures. Antiapoptotic Bcl-2 and Bcl-xL were upregulated, and proapoptotic Bax was downregulated in 3D cultures than in 2D cultures. Bar graphs depict densitometry quantitation of Bcl-2, Bcl-xL, and Bax protein expression normalized to β-actin. Data represent the means ± SD of three independent experiments. ^*^P < 0.05 and ^**^P < 0.01 versus the control.

An annexin V-FITC assay using flow cytometry was performed to evaluate the ability of doxorubicin to induce apoptosis. As shown in Figure 2A, an apoptosis assay showed a significant difference between 2D and 3D cultures with regard to the degree of apoptosis induction. The apoptosis rate to doxorubicin was 556.6 ± 4.5% (versus 1117.3 ± 18.1% for 2D) in EL4 cell spheroids and 121.4 ± 26.7% (versus 7180.5 ± 17.3% for 2D) in A20 cell spheroids in comparison to an untreated control condition (considered as 100%) grown during the same time. This indicated that the resistance to apoptosis induced by doxorubicin in both cell types was markedly increased in 3D cultures compared to 2D cultures.

EL4 cells after 3D culture for 10 days in AmCA hydrogels had significantly higher Bcl-2 and Bcl-xL protein levels (7.1-fold and 2.4-fold, respectively) and lower Bax protein levels (0.7-fold) compared to the 2D controls as assessed by western blotting (Figure 2B). This indicated that the activation of Bcl-2 family proteins (Bcl-2, Bcl-xL, and Bax), the key regulators of apoptosis, is implicated in the molecular mechanism by which the 3D microenvironment in AmCA hydrogels regulates apoptosis in lymphoma cells.

### 3D microenvironment in AmCA hydrogels facilitates tumor progression

As tumor cells grown in 3D models that efficiently reflect the 3D nature of the *in vivo* microenvironment are believed to have more malignant phenotypes than those in traditional 2D monolayer cultures, we hypothesized that the multicellular 3D lymphoma spheroids generated in AmCA hydrogels may show expedited progression compared to cells cultured in 2D. To evaluate the effects of the 3D microenvironment provided by AmCA hydrogels on the expression of molecules that play essential roles in tumor progression, we seeded EL4 cells at 1 × 10^5^ cells/ml into these hydrogels. On day 10, when multicellular spheroids of these cells were formed in 3D conditions, the expression of Notch receptor genes (Notch-1, -2, -3, and -4), vascular endothelial growth factor (VEGF), and matrix metalloproteinases (MMP-2 and MMP-9) was compared in the cells grown in 2D or 3D cultures.

**Figure 3:**
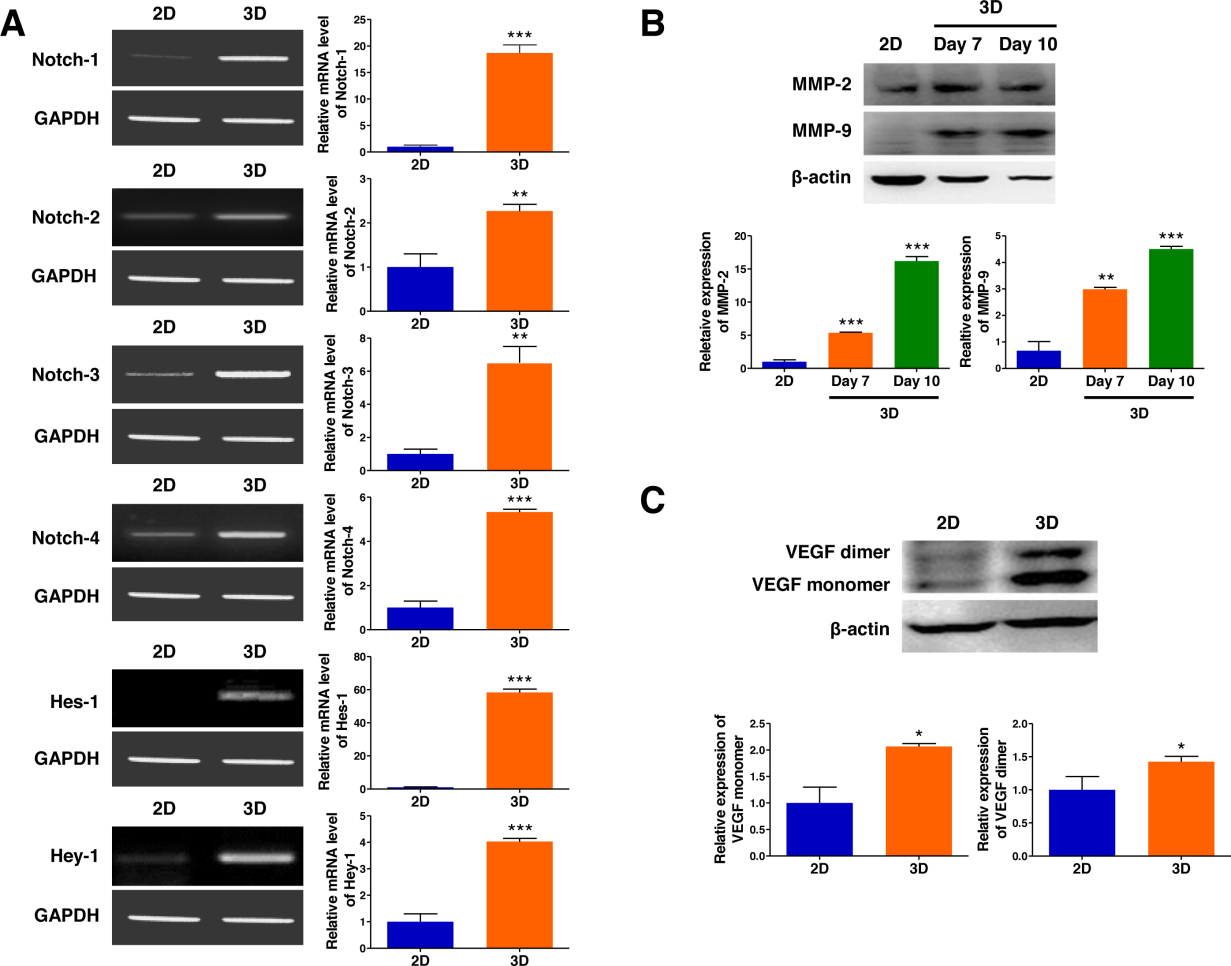
3D microenvironment in AmCA hydrogels enhances the expression of tumor aggressiveness factors in 3D lymphoma spheroids compared to their 2D cultures (**A**) RT-PCR demonstrates upregulated gene expression of Notch (Notch-1, -2, -3, and -4) and its downstream molecules (Hes-1 and Hey-1) important in tumor promotion and progression in 3D spheroids compared to 2D culture. Bar graphs depict densitometry quantitation of Notch, Hes-1, and Hey-1 mRNA expression normalized to GAPDH mRNA. Western blot demonstrates upregulated expression of (**B**) MMPs (MMP-2 and MMP-9) and (**C**) VEGF proteins important in tumor progression and metastasis in 3D spheroids compared to 2D culture. Bar graphs depict densitometry quantitation of MMP-2, MMP-9, and VEGF protein expression normalized to β-actin. Data represent the means ± SD of three independent experiments. ^*^P < 0.05, ^**^P < 0.01, and ^***^P < 0.001 versus the control.

Activation of the Notch signaling pathway, playing central roles in proliferation and progression of a variety of tumor cell types including lymphoma, was evaluated by RT-PCR to further explore the molecular mechanism by which the 3D microenvironment in AmCA hydrogels regulates tumor aggressiveness in lymphoma cells. As shown in Figure 3A, EL4 cells after 3D culture for 10 days in AmCA hydrogels expressed strikingly elevated gene transcript levels of Notch-1, -2, -3, and -4 (18.6-fold, 2.2-fold, 6.5-fold, and 5.4-fold, respectively) as well as Hes-1 and Hey-1 (58.3-fold and 4.0-fold, respectively), compared to the 2D controls. To explore the molecular mechanism by which the 3D microenvironment in AmCA hydrogels regulates malignancy in lymphoma cells, the expression levels of MMP and VEGF, critical molecules in tumor cell metastasis and angiogenesis, was determined by western blotting. As shown in Figure 3B, EL4 cells after 3D culture for 7 or 10 days in AmCA hydrogels exhibited strongly enhanced expression of MMP-2 (5.3-fold and 16.1-fold, respectively) and MMP-9 (2.9-fold and 4.5-fold, respectively). Additionally, EL4 cells after 3D culture for 10 days in AmCA hydrogels expressed robustly augmented expression of VEGF proteins (2.0-fold for the monomer and 1.4-fold for the dimer) compared to the 2D controls (Figure 3C).

**Figure 4:**
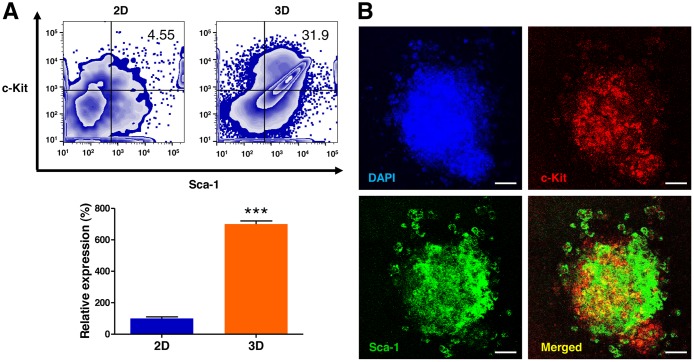
3D microenvironment in AmCA hydrogels augments the lymphoma stem cell population in 3D lymphoma spheroids compared to their 2D cultures (**A**) Flow cytometric analysis of EL4 cells for the expression of the lymphoma stem cell markers c-Kit and Sca-1. Percentages of the cell population expressing c-Kit^+^/Sca-1^+^ in EL4 cells cultured under 2D and 3D conditions are presented. Data represent the means ± SD of three independent experiments. ^***^P < 0.001 versus the control. (**B**) Confocal microscopic analysis of EL4 cells for the expression of c-Kit and Sca-1. Immunofluorescence images show the distribution features of lymphoma stem cells in the spheroids. Scale bar, 40 μm.

To determine whether 3D culture in AmCA hydrogels enhance the population of c-Kit^+^/Sca-1^+^ lymphoma stem cells, the cells were assessed by flow cytometry. As shown in Figure 4, the 3D microenvironment in AmCA hydrogels profoundly augmented the ratio of lymphoma stem cells by 700.3-fold compared to the control 2D cell culture.

### 3D microenvironment in AmCA hydrogels upregulates multidrug resistance-related gene expression

**Figure 5:**
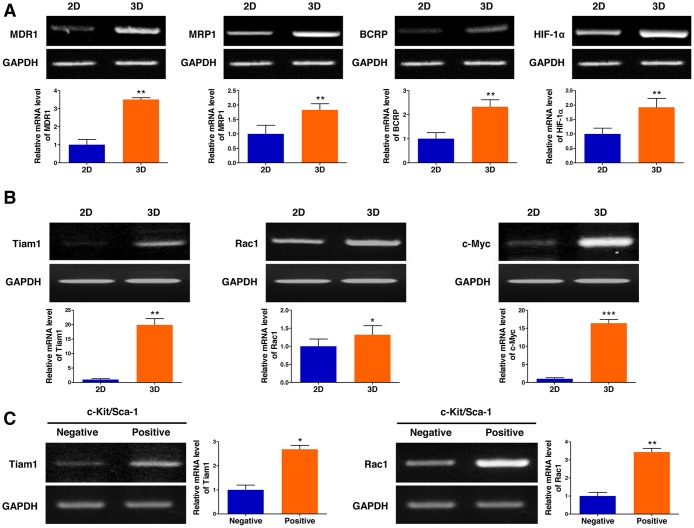
3D microenvironment in AmCA hydrogels elevates the expression of multidrug resistance-related genes in 3D lymphoma spheroids compared to their 2D cultures (**A**) RT-PCR demonstrates upregulated expression of MDR1, MRP1, BCRP, and HIF-1α genes important in promoting malignancy and multidrug resistance in 3D spheroids compared to 2D culture. Bar graphs depict densitometry quantitation of MDR1, MRP1, BCRP, and HIF-1α mRNA expression normalized to GAPDH mRNA. (**B**) RT-PCR demonstrates upregulated expression of Tiam1, Rac1, and c-Myc genes important in tumor progression and chemoresistance in 3D spheroids compared to 2D culture. Bar graphs depict densitometry quantitation of Tiam1, Rac1, and c-Myc mRNA expression normalized to GAPDH mRNA. (**C**) RT-PCR analysis of EL4 cells for the expression of Tiam1 and Rac1 genes by c-Kit^+^/Sca-1^+^ stem cells and c-Kit^-^/Sca-1^-^ non-stem cells. Bar graphs depict densitometry quantitation of Tiam1 and Rac1 mRNA expression normalized to GAPDH mRNA. Data represent the means ± SD of three independent experiments. ^*^P < 0.05, ^**^P < 0.01, and ^***^P < 0.001 versus the control.

Multidrug resistance remains a major obstacle to successful cancer chemotherapy. As tumor cells grown in 3D models that effectively reflect the 3D nature of the *in vivo* microenvironment are considered to have more malignant phenotypes than those in traditional 2D monolayer cultures, we hypothesized that the multicellular 3D lymphoma spheroids formed in AmCA hydrogels may display intensified chemoresistance-related gene expression. To investigate effects of the 3D microenvironment provided by AmCA hydrogels on the expression of multidrug resistance-related genes, we seeded EL4 cells at 1 × 10^5^ cells/ml into these hydrogels. On day 10, when multicellular spheroids were formed in 3D conditions, the expression of the following four important chemoresistance genes was compared in the cells grown in 2D or 3D cultures: (1) MDR1 which is also known as permeability glycoprotein (P-glycoprotein, P-gp) or ATP-binding cassette subfamily B member 1 (ABCB1), (2) multidrug resistance-associated protein 1 (MRP1, ABCC1), (3) breast cancer resistance protein (BCRP, ABCG2), and (4) hypoxia-inducible factor-1α (HIF-1α). Subsequently, we found that the expression of all the multidrug resistance-related genes tested, i.e., MDR1, MRP1, BCRP, and HIF-1α, were upregulated (3.5-fold, 1.8-fold, 2.3-fold, and 1.9-fold, respectively) in 3D-cultured EL4 cells (Figure 5A). Importantly, minimal expression of MDR1 and BCRP was observed in traditional 2D culture. These results suggest that drug resistance in cells grown in 3D AmCA hydrogel scaffolds could be attributed to increased drug efflux and tumor hypoxia.

To elucidate the molecular mechanism by which the 3D microenvironment in AmCA hydrogels augments multidrug resistance in lymphoma cells, the level of Tiam1 was assessed by RT-PCR. As shown in Figure 5B, expression of molecules involved in the Tiam1 signaling axis, Tiam1, Rac1, and c-Myc, was elevated (19.3-fold, 1.3-fold, and 16.3-fold, respectively) in 3D-cultured EL4 cells. These results suggest that the Tiam1/Rac1 signaling pathway is activated in the multicellular 3D lymphoma spheroids constructed within AmCA hydrogels. Multidrug resistance has also been suggested to be associated with tumor stem cells [23]. As we hypothesized that the lymphoma stem cells present in the multicellular 3D lymphoma spheroids constructed within AmCA hydrogels may be relevant to multidrug resistance, we explored effects of the 3D microenvironment provided by AmCA hydrogels on the expression of Tiam1 and Rac1 genes after isolating the EL4 lymphoma stem cells. Interestingly, the expression of Tiam1 and Rac1 genes was dramatically increased (2.6-fold and 3.4-fold, respectively) in the EL4 stem cells compared to EL4 non-stem cells (Figure 5C).

### Potential of the 3D cell culture to simulate tumor growth *in vivo*

**Figure 6:**
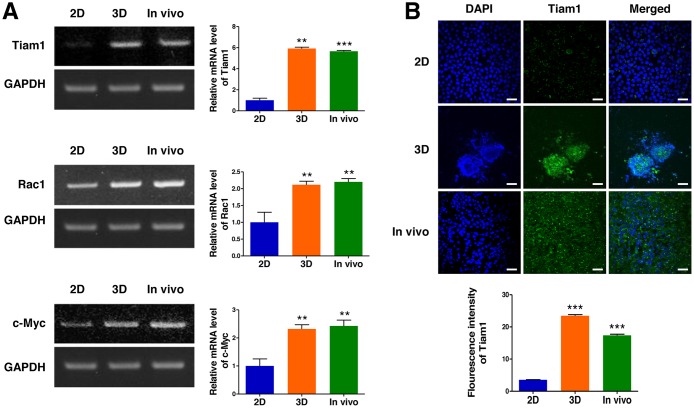
Expression of Tiam1, Rac1, and c-Myc in 2D- and 3D-cultured EL4 cells and their *in vivo* tumor tissue (**A**) RT-PCR demonstrates upregulated expression of Tiam1, Rac1, and c-Myc genes in 3D spheroids compared to 2D culture. EL4 cells expressed higher levels of Tiam1 in the 3D culture and fresh EL4 tumor than in the 2D culture, with no significant difference in the expression level of Tiam1/Rac1 between the 3D culture and fresh *in vivo* tumor. Bar graphs depict densitometry quantitation of Tiam1, Rac1, and c-Myc mRNA expression normalized to GAPDH mRNA. (**B**) Immunofluorescence images also show that EL4 cells expressed higher levels of Tiam1 in the 3D culture and fresh EL4 tumor than in 2D culture. The Tiam1 staining intensities were measured using ImageJ software. Data represent the means ± SD of three independent experiments. Scale bar, 40 μm. ^*^P < 0.05, ^**^P < 0.01, and ^***^P < 0.001 versus the control.

To explore the relevance of a 3D lymphoma cell culture in AmCA hydrogels to tumor growth *in vivo*, we compared excised tumor tissue with the 2D- and 3D-lymphoma cell cultures in terms of Tiam1, Rac1, and c-Myc expression. As shown in Figure 6A, the relative expression of Tiam1 mRNA in the EL4 cell spheroids generated in AmCA hydrogels and fresh EL4 tumor were 5.9-fold and 5.6-fold greater than the 2D-cultured EL4 cells, respectively. Moreover, consistent with the Tiam1 expression, Rac1 and c-Myc expression was much higher in the 3D-cultured EL4 cells (2.1-fold and 2.3-fold, respectively) and fresh EL4 tumor (2.2-fold and 2.4-fold, respectively) than the 2D-cultured EL4 cells (Figure 6A). These results were also confirmed by immunofluorescence staining of Tiam1 (Figure 6B). These findings confirm that a 3D cell culture in AmCA hydrogels is an exceedingly favorable system to represent *in vivo* tumor characteristics.

### Repression of Tiam1 expression prevents cell growth in 3D lymphoma spheroids

**Figure 7:**
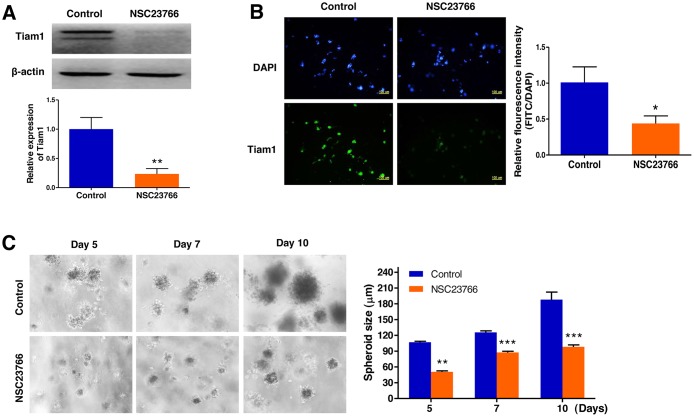
Repression of Tiam1 expression prevents cell growth in 3D lymphoma spheroids (**A**) Western blot and (**B**) immunofluorescence microscopy demonstrates the strong inhibition of Tiam1 expression by NSC23766 in 3D spheroids. (**C**) Phase contrast microscopy demonstrates a marked reduction in the size of EL4 cell spheroids by inhibition of endogenous Tiam1. Data represent the means ± SD of three independent experiments. ^*^P < 0.05, ^**^P < 0.01, and ^***^P < 0.001 versus the control.

To validate inhibition of Tiam1 expression by NSC23766, a specific inhibitor of the Tiam1/Rac1 signaling module, we performed a western blot assay and immunofluorescence microscopy. These analyses led us to confirm that Tiam1 expression was significantly downregulated by NSC23766 in 3D culture (Figure 7A and B). We next investigated the impact of the Tiam1/Rac1 inhibitor on the growth of EL4 cells cultured in 3D microenvironments using AmCA hydrogels by phase contrast microscopy (Figure 7C). Inhibition of endogenous Tiam1 resulted in a marked reduction in the size of EL4 cell spheroids. The average spheroid diameter of EL4 cells in the untreated control group was 105.0, 115.1, and 187.9 μm on day 5, 7, and 10, respectively. After Tiam1 inhibition, the average spheroid diameter was 50.7, 80.9, and 98.2 μm on day 5, 7, and 10, respectively, suggesting that Tiam1 plays a crucial role in the growth of EL4 cells (Figure 7C).

### Combined targeting of Tiam1 and Notch synergistically increases sensitivity to doxorubicin in the EL4 T and A20 B lymphoma cells

Despite significant advances in the treatment of lymphoma, drug resistance remains a major cause of treatment failure. To investigate the combined inhibitory effects of Tiam1 and Notch on resistance to doxorubicin in the 3D-cultured EL4 and A20 cells, we seeded EL4 and A20 cells at 1 × 10^5^ cells/ml into these hydrogels. On day 10, when the cell spheroids were formed in 3D condition, doxorubicin (1 μM) was treated in combination with Tiam inhibitor (100 μM NSC23766) or/and Notch inhibitor (5 μM DAPT) in the cells grown in 2D or 3D cultures.

**Figure 8:**
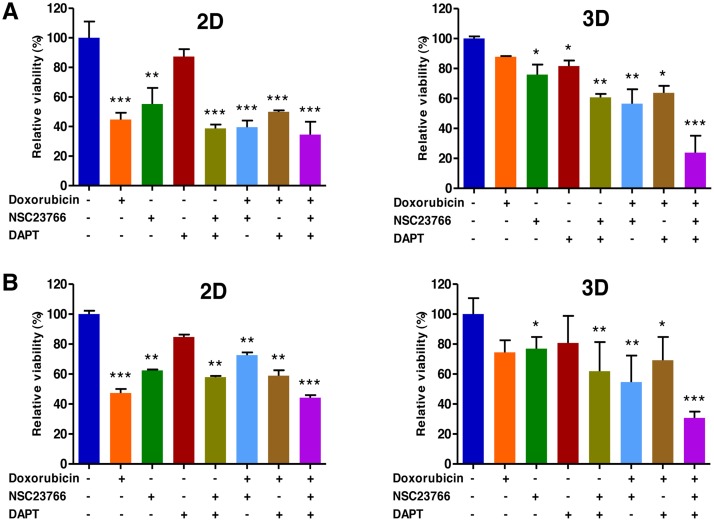
Combined targeting of Tiam1 and Notch synergistically increase sensitivity to doxorubicin in 2D- and 3D-cultured EL4 T (**A**) and A20 (**B**) B lymphoma cells. (A) WST-1-based colorimetric cell cytotoxicity assay demonstrates a significant difference between 2D and 3D cultures with regard to doxorubicin sensitivity. The levels of increased sensitivity to doxorubicin in the group pretreated with either the Tiam1/Rac1 inhibitor or the Notch inhibitor compared to the group treated with doxorubicin alone are significantly higher in 3D than in 2D condition in both cell types. The levels of increased sensitivity to doxorubicin in the group pretreated with a combination of Tiam1 and Notch inhibitors compared to the group pretreated with either the Tiam1/Rac1 inhibitor or the Notch inhibitor are much higher in 3D than in 2D condition in both cell types. Remarkably, the augmented sensitivity to doxorubicin by combined pretreatment with Tiam1 and Notch inhibitors is more prominent in 3D lymphoma spheroids compared to the 2D-cultured cells in both cell types. Data represent the means ± SD of three independent experiments. ^*^P < 0.05, ^**^P < 0.01, and ^***^P < 0.001 versus the control.

A WST-1-based colorimetric cell cytotoxicity assay was employed to evaluate the ability of antitumor agents to induce cytotoxicity. As shown in Figure 8A and B, a cell cytotoxicity assay showed a significant difference between 2D and 3D cultures with regard to antitumor agent sensitivity. In EL4 cells the rates of cell viability in 3D cultures to doxorubicin alone, NSC23766 alone, DAPT alone, doxorubicin pretreated with NSC23766, doxorubicin pretreated with DAPT, combination of NSC23766 with DAPT, and doxorubicin pretreated with combination of NSC23766 and DAPT were 87.8 ± 0.5% (versus 44.8 ± 4.5% for 2D), 75.9± 6.7% (versus 55.2 ± 10.9% for 2D), 81.7 ± 3.6 (versus 87.3 ± 5.0% for 2D), 60.8 ± 2.2% (versus 38.8 ± 2.5% for 2D), 56.6 ± 9.5% (versus 39.6 ± 4.4% for 2D), 63.8 ± 4.6% (versus 49.9 ± 1.0% for 2D), and 23.9 ± 11.2% (versus 34.5 ± 8.7% for 2D), respectively (Figure 8A). An untreated condition (considered as 100%) grown during the same time served as the control. A similar effect was also observed with A20 cell spheroids; the rates of cell viability in 3D cultures to doxorubicin alone, NSC23766 alone, DAPT alone, doxorubicin pretreated with NSC23766, doxorubicin pretreated with DAPT, combination of NSC23766 with DAPT, and doxorubicin pretreated with combination of NSC23766 and DAPT were 74.4 ± 8.1% (versus 47.3 ± 2.6% for 2D), 76.9± 7.7% (versus 62.4 ± 0.5% for 2D), 80.7 ± 18.0 (versus 84.7 ± 1.5% for 2D), 61.9 ± 19.4% (versus 57.9± 0.8% for 2D), 54.6 ± 17.6% (versus 72.6 ± 1.8% for 2D), 69.2 ± 15.5% (versus 58.9 ± 3.6% for 2D), and 30.7 ± 4.2% (versus 44.2 ± 1.6% for 2D), respectively (Figure 8B).

The levels of increased sensitivity to doxorubicin in the group pretreated with either the Tiam1/Rac1 inhibitor or the Notch inhibitor compared to the group treated with doxorubicin alone were significantly higher in 3D than in 2D condition in both cell types (Figure 8). The levels of increased sensitivity to doxorubicin in the group pretreated with a combination of Tiam1 and Notch inhibitors compared to the group pretreated with either the Tiam1/Rac1 inhibitor or the Notch inhibitor were much higher in 3D than in 2D condition in both cell types (Figure 8). The augmented sensitivity to doxorubicin by combined pretreatment with Tiam1 and Notch inhibitors was more prominent in 3D lymphoma spheroids compared to the 2D-cultured cells in both cell types (Figure 8).

## DISCUSSION

Unlike 2D cell culture, 3D cell culture has the potential to make an innovative breakthrough in the understanding of tumor biology, metastasis, oncotargets, biomarkers, drug discovery, and targeted therapies [24]. An accumulating body of evidence indicates that multicellular 3D spheroids fabricated in properly assembled hydrogels can serve as a favorable carcinoma model, and their spatial 3D organization offers collective properties of a cell population that may profoundly affect important tumor cell behavior such as tumor aggressiveness and chemotherapy resistance [25]. Despite an intense interest in 3D tumor models, there have been only a few attempts to develop 3D lymphoma culture systems [26]. Birgersdotter et al. [27] used self-assembling peptide-based hydrogels for the construction of a 3D Hodgkin lymphoma cell culture model and found that the 3D cell culture induced a more tumor-related gene expression profile compared to 2D cell culture. Caicedo-Carvajal et al. [28] tested the efficacy of a 3D lymphoma cell culture using polystyrene polymer scaffolds and demonstrated that the 3D culture model had a higher proliferative rate than the 2D culture model. Gravelle et al. [29] demonstrated that multicellular aggregates of follicular lymphoma cells, obtained by a simple 3D cell culture technique, show different cell properties such as proliferation, signaling pathway, cellular defenses, sensitivity to immune effectors, and resistance to doxorubicin and bendamustine compared to 2D cell culture. In addition, these multicellular aggregates of follicular lymphoma cells also exhibited different responses to antibody-based therapeutics between 3D and 2D cultures [25]. These results support the use of 3D multicellular aggregates of follicular lymphoma cells for the testing of therapeutic agents in follicular lymphoma. Recently, Tian et al. [30] synthesized integrin-specific ligand functionalized 3D hydrogel organoids of malignant B and T cell tumors using maleimide functionalized 4-arm polyethylene glycol and thiolated cross-linkers. They demonstrated that the 3D culture exhibited enhanced proliferation, clustering, and drug resistance to doxorubicin and a histone deacetylase inhibitor, panobinostat [30]. Furthermore, it was found that the 3D microenvironment in diffuse large B cell lymphomas upregulated the expression level of B cell receptor, which is involved in the survival of B lymphoma cells [30]. While these studies have provided evidence that lymphoma cells grown in 3D behave in a way reminiscent of the *in vivo* tissue microenvironment of malignant B and T cell lymphoma, further studies are necessary for a more comprehensive assessment of diverse aspects of potential 3D lymphoma models.

In this study, we established an efficient 3D T and B cell lymphoma culture model using AmCA hydrogels, and elucidated that increased resistance against antitumor agents in the 3D EL4 T and A20 B lymphoma models may be attributed to high levels of drug resistance gene expression, multiple perturbations in the apoptosis pathway, elevated levels of factors associated with tumor aggressiveness, hypoxia induction, augmented angiogenic potential, and enhanced tumor stemness. Importantly, the salient feature of our data is that combined treatment with inhibitors of Tiam1 and Notch was found to synergistically increase sensitivity to doxorubicin in EL4 and A20 cells than either treatment alone. It is also noteworthy that EL4 cells expressed higher levels of Tiam1 in the 3D culture and fresh EL4 tumor compared to the 2D culture, while there was no significant difference in the expression level of Tiam1/Rac1 between the 3D culture and fresh *in vivo* tumor. This suggests that our 3D lymphoma model precisely reflects the essential characteristics of an *in vivo* tumor including drug resistance, and thereby provides a preferential drug screening platform that can produce more clinically relevant results.

It has previously been reported that Tiam1 is highly expressed in lymphoma, leukemia, and many solid tumors including pancreatic, breast, bladder, lung, colorectal, gastric, liver, ovarian, prostate, and neck squamous cell cancer [17, 31, 32]. Evidence has been accumulating that Tiam1 plays a critical role in tumor initiation, promotion, and progression of various tumor cells including breast, colorectal, skin, and liver cancer [18, 19]. It was recently found that Tiam1 promotes proliferation, invasion, and metastasis in oral squamous cell carcinoma cells by binding with Semaphorin 4D [33]. It was also shown that Tiam1 expression is strongly associated with grade and outcome in ovarian carcinoma, and it may serve as a useful molecular marker for prognosis and clinical management [34]. Moreover, the Tiam1/Rac1 signaling axis contributes to tumorigenic progression in triple-negative breast cancer by mediating the acquisition of integrin-directed metastasis-associated tumor cell phenotypes [35]. Despite many studies on the role of Tiam1 in various types of tumors, surprisingly little information is available about the role of Tiam1 in lymphoma. Tiam1 expression was observed to be higher in extranodal NK/T-cell lymphoma tissue than the control [36]. Cordo-Russo et al. [21] showed that lymphoma cell lines resistant to chemotherapeutic agents presented a higher migratory capacity towards hyaluronan *in vitro* as well as a higher Tiam1 expression than the sensitive cell line, suggesting the possible implications of Tiam1 for the chemoresistance in lymphoma. Notably, we confirmed in the present study that Tiam1 is involved in chemoresistance against doxorubicin in EL4 T and A20 B lymphoma cells.

Multidrug resistance, a complex biological phenomenon whereby tumor cells become resistant to a wide spectrum of drugs with different structures or cellular targets, is a common cause of failure in chemotherapy. It is generally accepted that the overexpression of the multidrug resistance proteins of tumor cells such as MDR1, MRP1, BCRP, and HIF-1α mediate multidrug resistance, although its mechanism is not yet clear [37–39]. The hypoxic microenvironment of the tumor tissue as well as its related characteristics such as low nutrient supply and low pH have been suggested to upregulate the expression of multidrug resistance proteins through specific cellular signaling pathways [39–41]. We demonstrated that our lymphoma models efficiently mimic the real tumor microenvironment since it has been reported that the size of multicellular spheroids more than about 100 μm in diameter contain an internal hypoxic zone caused by limited distribution of oxygen, nutrients and metabolites, and a necrotic core [42, 43]. Although tumor cells grown in 2D culture have contributed tremendous amounts of knowledge regarding the mechanism of cancer, this system has unfortunately resulted in a 95% drug failure rate [44]. Thus, 3D models with customized microenvironments yield the greatest value in the vast field of biomedical research, especially including studies of tumor biology and establishment of therapeutic screens. Moreover, in antitumor drug testing, the 3D organization of the tumor mass and the complex 3D ECM network architecture have important advantages, as the 2D cultures do not effectively recapitulate the multidrug resistance-conducive environment [45, 46].

The Notch pathway is one of the most intensively studied candidate therapeutic targets for tumor cells, as Notch signaling is critical for cell proliferation, aggressiveness, and chemoresistance as well as stem cell propagation in diverse types of primary and metastatic tumors [47]. The molecular mechanisms underlying acquisition of chemoresistance coordinated by Notch signaling are believed to involve the induction of the epithelial-mesenchymal transition (EMT), formation of tumor stem cells, upregulated expression of MDR such as the MDR1, MRP1, BCRP, and HIF-1α, and the enhanced production of oncogenic microRNAs (miRNAs) [48–50]. Thus, many scientists are trying to develop targeted therapeutic strategies to inhibit the Notch pathway for the successful treatment of human malignancies.

Tumor stem cells, present in different types of malignancies, appear to share common features including a state of relative quiescence and a self-renewal capacity in the context of preferentially asymmetric divisions [51]. Furthermore, they are reported to be highly resistant to chemotherapeutic treatments or irradiation, possibly due to their high DNA repair capacity and to the expression of multidrug resistance [52]. Hence, development of novel therapies targeted at tumor stem cells holds great hope for the treatment of multidrug-resistant and metastatic tumors. Interestingly, they preferentially tend to grow in spheroid-like structures *in vitro* likely by alterations in their characteristics including loss of cell polarity or defective interaction with ECM components, although its exact mechanism remains elusive [53, 54]. Different types of matrices have also been used to promote their maintenance or differentiation [55–56]. Our data indicate that the AmCA hydrogels provide a favorable 3D culture platform for expansion of cells characterized by lymphoma stem cell-like features, and they can be very useful in the study of lymphoma stem cells as well as in the development of a new and potential therapeutic strategy for lymphoma.

In conclusion, we developed an effective *in vitro* 3D lymphoma cell culture system using AmCA hydrogel scaffolds that can imitate the *in vivo* 3D lymphoma microenvironment. Lymphoma cells grown in these 3D culture system exhibited biochemical and physiological features including (1) enhanced chemotherapy resistance, (2) suppressed rate of apoptosis, (3) upregulated expression of multidrug resistance-related genes (MDR1, MRP1, BCRP, and HIF-1α), (4) elevated levels of key molecules associated with tumor progression and malignancy including apoptosis-regulating molecules (Bcl-2, Bcl-xL, and Bax), Notch (Notch-1, -2, -3 and -4) and its downstream molecule (Hes-1 and Hey-1), VEGF, and MMPs (MMP-2 and MMP-9), and (5) enrichment of a lymphoma stem cell population by about 700-fold, compared to 2D-based conventional suspension cell culture. Importantly, Tiam1 as a potential biomarker of tumor initiation and progression, metastasis, and chemoresistance in many tumor cells was activated in our 3D lymphoma model. Remarkably, we identified two synergistic therapeutic oncotargets, Tiam1 and Notch, as a strategy to combat resistance against doxorubicin in EL4 T and A20 B lymphoma. Therefore, our data suggest that our 3D lymphoma model is a promising *in vitro* research platform to study lymphoma biology as well as screen new anti-lymphoma therapeutics.

## MATERIALS AND METHODS

### Cell line, culture maintenance, and materials

The biomaterials used for fabricating hydrogels were sodium alginate (W201502, Sigma-Aldrich, St. Louis, MO, USA), agarose (32802, high melting agarose, Affymetrix, Cleveland, OH, USA), PBS without CaCl_2_ and MgCl_2_ (D5652, Sigma-Aldrich), bovine serum albumin (A9647, BSA, Sigma-Aldrich), and marine collagen (MC) extracted from tilapia fish scales (Geltech, Busan, Korea). 4,6-Diamidino-2-phenylindole (DAPI) was purchased from Vector Laboratories (H-1200, Burlingame, CA, USA). For the tumor cell lines, EL4 cells (TIB-39™; a mouse thymic T cell lymphoma cell line) and A20 cells (TIB208™; a mouse B cell lymphoma cell line) were purchased from the American Type Culture Collection (ATCC, Manassas, VA, USA). Both cell types were cultured in RPMI-1640 medium (11875–093, Invitrogen, Carlsbad, CA, USA) containing 10% FBS, 100 IU/ml penicillin, and 100 μg/ml streptomycin at 37 °C in a 5% CO_2_ incubator. All cell culture reagents were obtained from Gibco/Thermo Fisher Scientific (Carlsbad, CA, USA) unless specified otherwise.

### Synthesis of hydrogels for 3D cell culture

AmCA hydrogels for 3D cell culture were prepared as previously described by our group [7]. Briefly, sodium alginate was dissolved at 50 mg/ml in deionized water by constant stirring overnight at room temperature to prepare a 5% alginate stock solution and then autoclaved before use. MC was completely dissolved by vortexing in nuclease-free water at room temperature to make a 25% stock solution. High melting point agarose was added at 20 mg/ml to deionized water, heated on a hot plate, and stirred occasionally until completely dissolved to make a 2% agarose stock solution. Then, 337 μl of cells resuspended in culture medium (1 × 10^5^ cells/ml) were mixed with 200 μl of 5% sodium alginate solution in a 1.5 ml microcentrifuge tube at room temperature. This solution was then combined with 400 μl of 25% MC stock solution at room temperature to obtain 10% MC/1% alginate solutions containing cells. These cell suspensions were then blended carefully with 62.5 μl of 2% agarose solution at 35–40°C to avoid cell damage. The final concentrations of agarose, MC, and alginate in the AmCA solution were 0.125%, 10%, and 1%, respectively.

For the gelation of hydrogel solutions containing cells, they were vortexed briefly, pipetted into 1 ml syringes, and finally incubated at 4°C for 5–10 min. The gelled hydrogels were then transferred to the wells of 24-well plates containing 1 ml of culture medium and incubated at 37°C for the desired time. Media were refreshed every 2 days.

### Cell spheroid-based antitumor drug test

To conduct antitumor drug tests against the cancer cell spheroids, doxorubicin (5927S, Cell Signaling, Danvers, MA, USA), docetaxel (01885, Sigma-Aldrich), resveratrol (R5010, Sigma-Aldrich), 5-FU (F6627, Sigma-Aldrich), vincristine (V8879, Sigma-Aldrich), and curcumin (C7727, Sigma-Aldrich) were used as antitumor agents in the present study. After spheroid formation, the drug-free culture media was replaced with 1 μM doxorubicin, 0.5 μM docetaxel, 100 μM resveratrol, 5 μM 5-FU, 1 μM vincristine, 10 μM curcumin, 100 μM Tiam1/Rac1 inhibitor (2221, NSC23766, Tocris Bioscience, Bristol, UK), and 5 μM N-[N-(3,5-difluorophenacetyl)-L-alanyl]-S-phenylglycine t-butyl ester (2634, DAPT, an inhibitor of the γ-secretase complex, Tocris Bioscience) in RPMI-1640. The efficacy of combination treatment of doxorubicin with either Tiam1 inhibitor or γ-secretase inhibitor, or with both agents was examined. Control groups of conventional 2D-cultured cells were treated with the same concentrations of these antitumor agents after reaching 60% to 80% confluence. After 24h treatment, cell viability was assessed using the EZ-Cytox assay (EZ-3000, Daeil Lab Service, Seoul, Korea) in 96-well microtiter plates by reading at an OD of 450 nm.

### *In vivo* EL4 lymphoma model

Male syngeneic C57BL/6 (H-2K^b^) mice were purchased from Dae Han Bio Link (Seoul, Korea). All animals were housed at 4–5 mice per cage and maintained under a 12 h light/dark cycle at a temperature of 20–22 °C with humidity 64 ± 15% in a filtered laminar air flow-controlled, specific pathogen-free facility. All mice were given irradiated food and sterile water *ad libitum*. Mice were allowed to adjust to their environment for 1 week and used at 8–10 weeks of age for implantation of tumor cells. 1 × 10^6^ EL4 cells were subcutaneously inoculated in the both left and right flanks of the mice (*n* = 5). Two weeks after injection, the mice were sacrificed, and the excised tumors were processed for experiments. Animal care and all experimental procedures were conducted in accordance with the “Guide for Animal Experiments edited by the Korean Academy of Medical Sciences.”

### Extraction of RNA and reverse transcription polymerase chain reaction for 3D hydrogels

For the tumor tissue, samples were transferred to tubes containing 1 ml TRIzol**^®^** Reagent (A33251, Invitrogen). Upon completion of the harvest procedure, the homogenates were transferred to empty RNase-free falcon tubes stored on ice. The homogenates were extracted with chloroform (400 μl), precipitated with isopropanol (400 μl), washed with ethanol (1 ml), and resuspended in 40 μl of distilled water. RNA concentrations and purity were determined by absorbance at 260 and 280 nm. Samples exhibiting an absorbance ratio (260/280) greater than or equal to 1.8 were used.

For the cell sample, the protocol used for RNA extraction was a modification of the RNeasy Mini Kit (Qiagen, Valencia, CA, USA) procedure. Briefly, AmCA hydrogels containing cells were diced in a mixture of 1.5 ml QG buffer (Qiagen) and 2 ml RLT buffer (74104, Qiagen). Samples were kept at room temperature until complete dissolution and then stored at -20 °C. After defrosting, samples were homogenized and supplemented with 2 ml 70% ethanol. Subsequent RNA purification steps were performed as described by the manufacturer, and RNA quantity and quality were assessed using a Nanodrop 2000 (ThermoFisher Scientific, Waltham, MA, USA).

First-strand cDNA was obtained by reverse transcription using 2 μg of total RNA. The reaction was conducted in 25 μl buffer containing 0.5 μg oligo(dT) 12–18 primer (Promega, Madison, WI, USA), 50 mM Tris-HCl (pH 8.3), 75 mM KCl, 3 mM MgCl_2_, 40 mM dithiothreitol, 0.5 mM deoxynucleotide triphosphate mixture (Promega), 10 U RNase inhibitor (Promega), and 200 U Moloney murine leukemia virus (MMLV) reverse transcriptase (Promega). After incubation at 37°C for 60 min, the reaction was stopped by heating at 70°C for 5 min. The obtained cDNA was used as a template for PCR amplification using the gene-specific primers. Primer sequences are shown in Table 1. PCR amplification of cDNA was performed in an automated thermal cycler (PC 320, Astec, Osaka, Japan) in a final volume of 25 μl containing 4 μl cDNA solution, 20 mM Tris-HCl (pH 8.4), 50 mM KCl, 1.5 mM MgCl_2_, 0.1% Triton X-100, 0.2 mM deoxynucleotide triphosphate mixture (Promega), 0.5 pmol of each primer, and 5 U Taq DNA polymerase (M3001, Promega). Amplified products were analyzed by electrophoresis in 2% agarose gel and visualized by ethidium bromide staining under UV light. Band intensities of PCR products were measured using an image analysis program (MetaMorph, Universal Imaging Corporation, Downingtown, PA, USA). Results were expressed as ratios versus GAPDH mRNA amplified from the same cDNA samples.

**Table 1:** RT-PCR primer names and their sequences

Gene name	Forward (5’-3’)	Reverse (5’-3’)
Notch-1	CCCAGCAGGTGCAGCCACAG	GGTGATCTGGGACGGCATGG
Notch-2	ATGTGGACGAGTGTCTGTTGC	GGAAGCATAGGCACAGTCATC
Notch-3	ACACTGGGAGTTCTCTGT	GTCTGCTGGCATGGGATA
Notch-4	AAGCGACACGTACGAGTCTGG	ATAGTTGCCAGCTACTTGTGG
Hes-1	CCAGCCAGTGTCAACACGA	AATGCCGGGAGCTATCTTTCT
Hey-1	TCTCCCTTCACCTCACTGCT	CACGCCACTATGCTCAATGT
MDR1	CATTGGTGTGGTGAGTCAGG	CTCTCTCACCAACCAGGGTG
MRP1	GTTCCCTCCGCATGAACTTG	CTGGCTCATGCCTGGACTCTG
BCRP	TCTCCTTGCCAGATAAGAGGGG	TGCCTCAAGAGACAGGCAAA
HIF-1α	AGCCCTAGATGGCTTTGTGA	TATCGAGGCTGTGTCGACTG
Tiam1	AACTTCACAAGTTACACCCACTC	CAGAAACCCCAGGCCAAACA
Rac1	AACCTGCCTGCTCATCAGTT	TGACAGCACCGATCTCTTT
c-Myc	AAGGGAAGACGATGACGG	TGAGAAACCGCTCCACATA
GAPDH	TGGAGA AACCTG CCAAGTATG	TTGTCATACCAGGAA ATGAGC

### Western blot analysis

To determine protein expression levels, EL4 cells cultured in 2D with or without treatment with Tiam1/Rac1 inhibitor (NSC23766) were pelleted by centrifugation at 2,000 rpm at 4°C for 5 min. After washing the hydrogels with ice cold PBS, EL4 cells cultured in 3D AmCA hydrogel for 7 and 10 days with or without treatment with Tiam1/Rac1 inhibitor (NSC23766) were harvested and washed twice with ice cold PBS. Then, the cells were incubated in ice-cold RIPA lysis buffer containing a cocktail of protease inhibitors (0.1 mM phenyl methane sulfonyl fluoride, 5 mg/ml aprotinin, 5 mg/ml pepstatin A, and 1 mg/ml chymostatin (9806S, Cell Signaling Technology, Denver, MA, USA) for 30 min for 2D-cultured cells and 3 h for 3D-cultured cells on ice. During incubation in lysis buffer, cell lysates were vortexed at each 10 min interval and then centrifuged at 13,000 rpm for 30 min at 4°C. After centrifugation, supernatants were collected as whole cell extracts. Protein concentration was determined using the bicinchoninic acid (BCA) protein assay (B9643, Sigma-Aldrich). Equal protein concentrations from each sample were mixed with Laemmli sample buffer, loaded, and then electrophoresed by sodium dodecyl sulfate-polyacrylamide gel electrophoresis (SDS-PAGE) on 8–12% (v/v) resolving gel and blotted onto a polyvinylidene fluoride membrane (Millipore, Bedford, MA, USA) via semi-dry transfer (IPVH00010, Bio-Rad, Hercules, CA, USA). The membrane was incubated in blocking buffer (5% BSA) and probed with mouse anti-MMP-2 (sc-13594), anti-MMP-9 (sc-12759), anti-Bcl-2 (sc-7382), anti-Bcl-xL (sc-7195), and anti-Bax (sc-20067) antibodies as well as rabbit anti-VEGF (sc-152) and anti-Tiam1 (sc-872) antibodies (all from Santa Cruz Biotechnology, Santa Cruz, CA, USA). The blots were then incubated with peroxidase-conjugated goat anti-mouse and anti-rabbit secondary antibodies (7076 and 7074, respectively, Cell Signaling Technology). Bound antibody was detected using enhanced chemiluminescence (ECL, Super Signal West Pico Chemiluminescent Substrate kit, Pierce, Rockford, IL, USA) following the manufacturer's instructions. Images were captured and quantified with a LAS-3000 imaging system (Fujifilm, Tokyo, Japan). Intensities of the bands were digitized using MultiGauge software (Fujifilm, Science Lab 2005, Japan).

### Sensitivity test to antitumor agents

After EL4 cells were cultured in 2D and in 3D for 10 days, cells were treated in serum-free media with doxorubicin (1 μM) alone or in combination with pretreatment of Tiam1/Rac1 inhibitor (100 μM NSC23766), Notch inhibitor (5 μM DAPT), or both for 24 h. To determine cell viability, the colorimetric WST-1 conversion assay (EZ-Cytox Assay Kit, Daeil Lab Service) was used. In brief, WST-1 reagent (20 μl) was added to each well, after which cells were incubated for 2 h in a humidified incubator at 37°C under 5% CO_2_. The absorbance of the formazan dye, generated by the reaction between dehydrogenase and WST-1 in metabolically active cells, was measured using a microplate reader (Tecan, Männedorf, Switzerland) at 450 nm according to the manufacturer's instructions. Percent cell viability was calculated. The morphology and size of cell spheroids were assessed at desired time points under a phase contrast microscope. All experiments were performed at least thrice.

### Confocal laser scanning microscopy

For confocal laser scanning microscopic analysis, EL4 cells (1 × 10^5^ cells/ml) were encapsulated in AmCA hydrogels, and cultured at 37°C in a 5% CO_2_ humidified incubator for 10 days. Cultured cells in 2D and hydrogels, and 5 μm-thick, frozen sections of *in vivo* tumor were washed with PBS and then fixed with cold 4% paraformaldehyde in 0.1 M phosphate buffer for 20 min. The fixative was then removed by washing the hydrogels three times for 5 min with cold PBS followed by permeabilization with 0.1% Triton X-100 in PBS for 5 min. After washing with cold PBS, they were incubated with 2% BSA (Sigma-Aldrich) for 1 h at room temperature. Excess solution was then removed and samples were incubated overnight at 4°C with PE-labeled anti-CD117 (c-Kit) and FITC-labeled anti-Sca-1 monoclonal antibodies (mAbs, 553355 and 557405, respectively, BD Biosciences, San Jose, CA, USA), or anti-Tiam1 antibody (sc-872, Santa Cruz Biotechnology), rinsed in cold PBS, and mounted on glass slides using Vectashield^®^ containing DAPI (Vector Laboratories). Cell fluorescence was observed using a confocal laser scanning microscope (FV1000 IX81, Olympus, Tokyo, Japan).

### Flow cytometry

Exposure of phosphatidylserine on the cell surface in apoptotic cells was detected with Annexin V-FITC Apoptosis Detection Kit (556570, BD Biosciences) according to the manufacturer's instructions. In brief, cells were incubated with doxorubicin at a concentration of 1 μM for 24 h. Apoptotic response assessment was detected by staining with fluorescein isothiocyanate (FITC)-labeled annexin V using FACSCanto II flow cytometer (BD Biosciences). At least 1 × 10^5^ cells were used for each assessment. To obtain cell suspension prior to flow cytometry analysis, the hydrogel debris was removed from cells by filtering the cell-polymer suspension through a 70-μm cell strainer (93070, SPL Life Sciences, Gyeonggi-do, Korea) to eliminate the hydrogel debris and all remaining cell clumps. Collected cells were washed twice with ice-cold PBS containing 1% FBS and 0.1% sodium azide, and incubated with FITC-conjugated Annexin V and propidium iodide (PI) for 20 min at room temperature in the dark. After staining, an electronic gate was set on the lymphoma cells using forward and side scatter characteristics.

To detect lymphoma stem cell population, EL4 cells were harvested from the 3D AmCA hydrogel cultured for 10 days by pipetting, washed with the culture medium containing 1% BSA and 0.1% sodium azide, and filtered through a 70-μm cell strainer (SPL Life Sciences). Phenotypic analysis of cell surface marker expression was performed by flow cytometry. Briefly, cells were washed twice with HBSS, and resuspended in the cell-staining buffer. Cells were immunostained for cell surface markers by incubating them for 30 min with PE-Cy7-labeled anti-c-Kit (565595, BD Biosciences), and APC-labeled anti-Sca-1 mAbs (108111, BioLegend, San Diego, CA, USA). Cells were washed twice and then resuspended in 50 μl of cell-staining buffer. For control, 2D-cultured cells were used. FACS analysis was performed using a FACS Canto-II flow cytometer (BD Biosciences). In all the experiments, the forward scatter and the side scatter were adjusted for each sample using appropriate unstained cells to eliminate dead cells and debris. Autofluorescence signals were eliminated by adjusting the signal outputs from designated channels, and the sensitivity was adjusted to collect a gated population of cells. Flow cytometry data were analyzed using FlowJo 10.3.0 (Tree Star, Ashland, OR, USA).

### Fluorescence-activated cell sorting

To disperse cells in a single cell suspension from the hydrogel matrix, the 10-day-AmCA hydrogels with embedded cells was thoroughly mixed with RPMI-1640 medium using a pipette. The cell-polymer suspension was washed with the culture medium and filtered through a 70-μm cell strainer (SPL Life Sciences) to eliminate the hydrogel debris and all remaining cell clumps. After washing cells with PBS at least twice, the cells were resuspended at a concentration of 4–5 million/ml. The cells were incubated with PE-CY7-labeled anti-c-Kit (558163, BD Biosciences) and APC-labeled anti-Sca-1 (557405, BD Biosciences) mAbs while agitating in an orbital shaker at 200 rpm for 45 min at 4°C in staining buffer containing PBS with 1% BSA and 0.1% sodium azide, washed twice in a wash buffer, and resuspended in a sort buffer containing PBS with 1% BSA. Then, the cells were sorted through a 100 μm nozzle at a sheath pressure of 20 psi and a drop drive frequency of 30 kHz into c-Kit^+^/Sca-1^+^ and c-Kit^-^/Sca-1^-^ cell populations on the FACSAria III cell sorter (BD Biosciences) using FACSDiva software. A highly pure sorting modality (4-way purity sorting for FACSAria III) was chosen. The flow rate during the sorting was approximately 8000 events/sec. The appropriate forward scatter and side scatter gating were used to isolate viable cells. Gates were set with reference to negative controls. The sorting speed was adjusted to ensure sorting efficiency above 90%. Sorted cells were collected in 5 ml polypropylene tubes containing 1 ml collection medium (RPMI 1640 media supplemented with 20% FBS).

## Abbreviations

AIDS: acquired immune deficiency syndrome
2D: 2-dimensional
3D: 3-dimensional
5-FU: 5-fluorouracil
ABCB1: ATP-binding cassette subfamily B member 1
AmCA: alginate/marine collagen/agarose
ATCC: American Type Culture Collection
BCRP: breast cancer resistance protein
BSA: bovine serum albumin
CLL: chronic lymphocytic leukemia
DAPI: 4,6-diamidino-2-phenylindole
DAPT: N-[N-(3,5-difluorophenacetyl)-L-alanyl]-S-phenylglycine t-butyl ester
ECL: enhanced chemiluminescence
ECM: extracellular matrix
EMT: epithelial-mesenchymal transition
FITC: fluorescein isothiocyanate
GDP: guanosine diphosphate
GTP: guanosine triphosphate
HIF-1α: hypoxia -inducible factor-1α
MC: marine collagen
MDR1: multidrug resistance protein 1
MMLV: murine leukemia virus
MMP: matrix metalloproteinases
P-gp: permeability glycoprotein
PP2A: protein phosphatase 2A
SDS-PAGE: sodium dodecyl sulfate-polyacrylamide gel electrophoresis
Tiam1: T-cell lymphoma invasion and metastasis 1
VEGF: vascular endothelial growth factor
